# Guidelines for Minimizing Bleeding Risk During Bronchoscopic Procedures

**DOI:** 10.3390/arm94040044

**Published:** 2026-06-30

**Authors:** Adam Barczyk, Anna Andrychiewicz, Małgorzata Czajkowska-Malinowska, Katarzyna Górska, Bartosz Hudzik, Piotr Korczyński, Rafał Krenke, Wojciech Naumnik, Wojciech J. Piotrowski, Cezary Piwkowski, Jerzy Soja, Artur Szlubowski, Jerzy Windyga, Joanna Zając, Filip Mejza

**Affiliations:** 1 Department of Pneumology, School of Medicine in Katowice, Medical University of Silesia, 40-635 Katowice, Poland; 2 Department of Endoscopy, University Hospital, Jagiellonian University Medical College, 30-688 Krakow, Poland; 3 Department of Lung Diseases and Respiratory Failure with the NWM Sub-Unit and the Sleep Breathing Disorders Sub-Unit, Kuyavian-Pomeranian Center of Pulmonology, 85-326 Bydgoszcz, Poland; 4 Department of Pulmonary Diseases, Internal Medicine, Thoracic Oncology and Transplantology, National Medical Institute, Ministry of the Interior and Administration, 02-507 Warsaw, Poland; drkpgorska@gmail.com (K.G.); drkorczynski@gmail.com (P.K.); 5 Third Department of Cardiology, Silesian Center for Heart Disease, Medical University of Silesia, 41-800 Zabrze, Poland; 6 Department of Cardiovascular Disease Prevention, Medical University of Silesia, 41-902 Bytom, Poland; 7 Department of Internal Medicine, Pulmonary Diseases, and Allergy, Medical University of Warsaw, 02-091 Warsaw, Poland; 8 1st Department of Lung Diseases, Lung Cancer, and Internal Diseases, Medical University of Bialystok, 15-540 Bialystok, Poland; 9 Department of Pneumology, Medical University of Lodz, 90-419 Lodz, Poland; wojciech.piotrowski@umed.lodz.pl; 10 Department of Thoracic Surgery, Poznan University of Medical Sciences, 60-512 Poznan, Poland; 11 Department of Pulmonology, 2nd Department of Internal Medicine, Jagiellonian University Medical College, 30-688 Krakow, Poland; 12 Endoscopy Unit, St. John Paul II Hospital, 31-202 Krakow, Poland; artur.szlubowski@gmail.com; 13 Bronchoscopy Unit, Klara Jelska’s Pulmonary Hospital, 34-500 Zakopane, Poland; 14 Department of Hemostasis Disorders and Internal Medicine, Institute of Hematology and Transfusion Medicine, 02-776 Warsaw, Poland; jerzy.windyga@gmail.com; 15 Department of Epidemiology and Preventive Medicine, Division of Hygiene and Dietetics, Jagiellonian University Medical College, 31-034 Krakow, Poland; 16 Department of Epidemiology and Preventive Medicine, Division of Epidemiology, Jagiellonian University Medical College, 31-034 Krakow, Poland

**Keywords:** bronchoscopy, bleeding risk, guidelines

## Abstract

**Highlights:**

- This article presents recommendations aimed at reducing the risk of bleeding during bronchoscopy.
- The document was developed by a working group convened by the Polish Respiratory Society.
- 13 recommendations/good clinical practice points were developed addressing bronchoscopy in patients with thrombocytopenia, abnormal activated partial thromboplastin time or international normalized ratio results, and in those receiving antiplatelet agents, oral anticoagulants, or low-molecular-weight heparin.

**Abstract:**

This article presents recommendations aimed at reducing the risk of bleeding during bronchoscopy. The document was developed by a working group convened by the Polish Respiratory Society, which included pulmonologists experienced in bronchoscopic procedures, an anesthesiologist, a thoracic surgeon, a cardiologist, a hematologist, a nurse, and methodologists. Clinical questions were formulated according to the PICO (Population, Intervention, Comparison, Outcome) framework, followed by a systematic literature search and critical appraisal of the selected studies. Based on these data, 13 recommendations/good clinical practice points were developed addressing bronchoscopy in patients with thrombocytopenia, abnormal activated partial thromboplastin time or international normalized ratio results, and in those receiving antiplatelet agents, oral anticoagulants, or low-molecular-weight heparin.

**Summary of Recommendations**

**Recommendations**

Strength/Quality of Evidence *
We suggest a minimum platelet count of ≥20,000/μL for safe performance of bronchoscopy or bronchoalveolar lavage (without biopsy).Conditional recommendation/low quality of evidenceIn patients scheduled for biopsy procedures (EBB, EBUS-TBNA, TBLB, TBLC), the platelet count should be ≥50,000/μL.Conditional recommendation/very low quality of evidenceAvailable scientific evidence and clinical practice do not support the routine assessment of INR and APTT in patients scheduled for bronchoscopy who are not receiving anticoagulant therapy, have no comorbidities requiring coagulation testing, and have no personal history suggestive of a bleeding tendency.Good practice pointAssessment of clinical risk factors for bleeding is essential to evaluating the risk of hemorrhagic complications during bronchoscopy.Good practice pointIn patients taking low-dose aspirin, discontinuation prior to bronchoscopy, BAL, EBB, EBUS-TBNA, TBLB, or TBLC is not required.Strong recommendation/moderate quality of evidenceIn patients requiring dual antiplatelet therapy, i.e., prophylactic aspirin combined with a P2Y12 receptor inhibitor (clopidogrel, prasugrel, or ticagrelor), an individualized assessment is recommended to weigh the benefits of bronchoscopy against the risks of procedural bleeding and stent thrombosis resulting from interruption of antiplatelet therapy.Conditional recommendation/very low quality of evidenceAlthough high-quality data specific to bronchoscopic procedures are lacking, multiple guidelines offer recommendations for the periprocedural management of patients receiving oral anticoagulants. Adherence to these recommendations is advised.Good practice pointIn patients currently taking oral anticoagulants: – bronchoscopy or bronchoscopy with BAL may be performed; – in patients receiving DOACs, the procedure should ideally be scheduled at the drug’s trough level (typically 12 or 24 h after the last dose); – in patients receiving VKAs, INR should be checked prior to the procedure; – biopsy procedures are not recommended.Good practice pointIf temporary interruption of anticoagulant therapy is planned: – the recommended timing for DOAC discontinuation prior to bronchoscopy with a planned biopsy is presented in the table in the full guideline; – VKAs should be discontinued 5 days before the procedure, and INR should be checked on the day of the procedure. Good practice pointRoutine heparin bridging is not recommended during the periprocedural period in patients whose oral anticoagulant therapy has been withheld, except in selected cases with a very high thromboembolic risk (see table in the full guideline).Good practice pointFor bronchoscopy without biopsy, the procedure may be performed while the patient is receiving prophylactic-dose LMWH.Good practice pointFor bronchoscopy with biopsy, it is suggested that LMWH be withheld prior to the procedure for 12 h when administered at a prophylactic dose, and for 24 h when administered at a therapeutic dose.Good practice pointResumption of LMWH after the procedure: if no bleeding complications occurred during the procedure, or if bleeding was adequately controlled, the next dose of LMWH may be administered after ≥8 h.Good practice point* Strength of recommendations and quality of evidence are based on the Grading of Recommendations Assessment, Development and Evaluation (GRADE) approach. Good practice points are based on extrapolation of data from non-bronchoscopy guidelines, narrative reviews, and the clinical experience of the panel members.

**Principles for Guideline Application and Implementation**

These recommendations are intended to support, but not replace, individual clinical judgment, which must always remain the foundation of clinical decision-making. In all cases, the potential benefits of the bronchoscopic procedure should be carefully weighed against the associated risks. Patient preferences should also be considered as part of the decision-making process.

## 1. Methods

Bronchoscopy is a fundamental procedure in the diagnosis and treatment of pulmonary diseases. One of its potential complications is localized bleeding. Patients at particularly increased risk of bleeding include those with iatrogenic coagulation disorders related to the use of antiplatelet or anticoagulant drugs. The selection and periprocedural management of such patients pose clinical challenges and uncertainty. To address this, at the initiative of the President-Elect of the Polish Respiratory Society (in Polish, Polskie Towarzystwo Chorób Płuc [PTChP]), a Working Group was convened to develop recommendations aimed at minimizing the risk of bleeding during bronchoscopic procedures.

### 1.1. Scope of the Guideline

A range of diagnostic and therapeutic procedures can be performed during bronchoscopy. This guideline focuses on the most commonly used diagnostic interventions in routine clinical practice:

(1) Collection of bronchial washings for microbiological, cytological, and other tests;
(2) Bronchoalveolar lavage (BAL);
(3) Endobronchial biopsy (EBB);
(4) Transbronchial needle aspiration (TBNA), most commonly performed under endobronchial ultrasound guidance (EBUS), i.e., EBUS-TBNA;
(5) Transbronchial lung biopsy using forceps (TBLB) and cryoprobe (TBLC).

These guidelines apply to all patients undergoing the above bronchoscopic procedures. This document does not apply to patients undergoing rigid bronchoscopy nor therapeutic bronchoscopic procedures.

### 1.2. Intended Users

This guideline is intended for pulmonologists, thoracic surgeons, and physicians in specialty training in these fields. It is also relevant for physicians from other specialties—such as anesthesiology, internal medicine, intensive care, and oncology—who perform bronchoscopic procedures. Additionally, the recommendations apply to nurses working in pulmonary wards and endoscopy units involved in the care of patients undergoing bronchoscopy.

### 1.3. Working Group

The Working Group that developed this guideline included pulmonary specialists with extensive experience in bronchoscopy, as well as a thoracic surgeon, cardiologist, hematologist, anesthesiologist, critical care specialist, procedural nurse, and methodologists.

### 1.4. Guideline Development

The guideline was developed in accordance with the principles of evidence-based medicine (EBM), incorporating some elements of the GRADE (Grading of Recommendations Assessment, Development and Evaluation) methodology [1].

Five key clinical questions were formulated:

1. In patients with abnormal platelet count, is it safe to perform bronchoscopy, BAL, EBB, EBUS-TBNA, TBLB, and TBLC? What is the minimum platelet count that allows these procedures to be performed safely?
2. In patients with abnormal coagulation parameters—specifically, prolonged prothrombin time (PT), expressed as international normalized ratio (INR), and activated partial thromboplastin time (APTT)—is it safe to perform bronchoscopy, BAL, EBB, EBUS-TBNA, TBLB, and TBLC? What are the maximum acceptable INR and APTT values that allow these procedures to be performed safely?
3. In patients taking 1 or 2 antiplatelet agents, is it safe to perform bronchoscopy, BAL, EBB, EBUS-TBNA, TBLB, and TBLC?
4. In patients receiving oral anticoagulants, is it safe to perform bronchoscopy, BAL, EBB, EBUS-TBNA, TBLB, and TBLC? If not, when should the anticoagulant be discontinued prior to the procedure, and when should it be resumed afterward?
5. In patients receiving low-molecular-weight heparin (LMWH) at prophylactic or therapeutic doses, is it safe to perform bronchoscopy, BAL, EBB, EBUS-TBNA, TBLB, and TBLC? If not, when should low-molecular-weight heparin (LMWH) be discontinued before the procedure, and when should it be resumed afterward?

Based on these key questions, a list of detailed clinical questions was developed and is presented in Supplementary Material File S1. These questions served as the basis for the literature search. For Question 4, the original formulation and literature search focused only on direct oral anticoagulants (DOACs). Following panel discussion, the clinical question was expanded to include vitamin K antagonists (VKAs), and the literature search was updated accordingly.

Given the scope of the guideline, the only primary outcome assessed was bleeding during bronchoscopy. For the purpose of this guideline, a bleeding severity scale was developed based on the most commonly used classifications in the literature (Table 1) [2,3,4,5]. The table also includes weights assigned to each bleeding severity grade as outcome measures.

Based on the detailed clinical questions, a systematic literature search of PubMed and EMBASE databases was conducted in March 2025 without restrictions on the start date. The search strategy combined free-text terms with controlled vocabulary from the MeSH and EMTREE thesauri. Studies relevant to the clinical questions were included regardless of methodological quality. Additional publications, including guidelines and secondary studies (systematic reviews, meta-analyses, or review articles), were also included. The detailed search strategy is described in Supplementary Material File S2. After duplicate removal, the remaining records were independently screened by 2 authors (FM and JZ). Any discrepancies were resolved through discussion. The database search was supplemented by manual screening of the reference lists of included publications, including identified guidelines, as well as relevant scientific society websites. The overall quality of the available evidence was assessed according to the GRADE classification. Primary and secondary studies were independently graded by 2 authors (FM and JZ) according to study or document type; however, no formal quality assessment process was applied to these publications. An exception was made for the guidelines, which were appraised using the AGREE II Global Rating Scale. In the absence of direct evidence, indirect data were considered when deemed relevant. Existing English-language guidelines on bronchoscopy were also reviewed [6,7,8,9,10,11,12,13,14]; two documents available only in Chinese were excluded [15,16]. Additionally, given the scope of the guideline, documents addressing the use of anticoagulants and antiplatelet agents, as well as perioperative management, were also considered. These included the most recent guidelines from the American College of Chest Physicians (ACCP) on anticoagulant therapy and from the British Society for Haematology on bleeding risk assessment prior to invasive procedures [17,18]. A synthesis of the scientific evidence related to each clinical question, including an assessment of evidence quality, was prepared and presented to the panel members. Based on the available evidence, the panel members formulated individual recommendations during joint meetings. Recommendations were prepared based on evidence synthesis and further refined through discussion until full consensus was reached among all panel members. No formal consensus method was used.

In accordance with the GRADE methodology, recommendations were classified as either strong or weak (conditional) (Table 2), and the quality of the supporting evidence was rated as high, moderate, low, or very low (Table 3). In the absence of high-quality direct evidence, good practice points were formulated by extrapolating from non–bronchoscopy-related guidelines, narrative reviews, and the clinical experience of the panel members.

### 1.5. Comments on the Risk–Benefit Balance

This document does not evaluate the risk–benefit balance for each clinical question individually. Instead, it focuses on assessing bleeding risk and the feasibility of safely performing the procedure. This approach reflects the fact that the potential benefit of bronchoscopy depends on the clinical indication (e.g., diagnostic value from obtaining a histopathological sample). Furthermore, in certain clinical scenarios, decision-making must also consider the potential cardiovascular risks associated with interrupting or modifying antiplatelet or anticoagulant therapy.

Therefore, the decision to proceed with bronchoscopy should always be individualized, made in collaboration with a well-informed patient, and, when appropriate, based on a consultation with other specialists (e.g., a cardiologist or hematologist).

### 1.6. Risk of Bleeding During Bronchoscopy

Bleeding during bronchoscopy occurs in 0.2–5% of cases; however, the risk of severe bleeding is much lower, estimated at around 0.031% [6,19,20,21]. Several factors influence bleeding risk. Key determinants include:

(1) Patient-related factors (e.g., coagulation status, use of antiplatelet or anticoagulant agents, and other bleeding risk factors);
(2) Location and type of structural lesions;
(3) Scope and characteristics of the procedure (Table 4) [19].

## 2. Recommendations

### 2.1. Question 1: In Patients with Abnormal Platelet Count, Is It Safe to Perform Bronchoscopy, BAL, EBB, EBUS-TBNA, TBLB, and TBLC? What Is the Minimum Platelet Count That Allows These Procedures to Be Performed Safely?

#### 2.1.1. Discussion of Scientific Evidence

Primary data: Several observational studies were identified, most of which included relatively small patient cohorts. It should be noted that significant confounding factors were present in many of these studies. In particular, a substantial proportion of patients received platelet transfusions, limiting the ability to draw reliable conclusions regarding platelet count thresholds associated with bleeding risk. One retrospective analysis described 2053 bronchoscopic procedures without biopsy, performed in 1711 patients with cancer. Among these patients, 990 had a platelet count > 100,000/μL (control group), 301 had 50–100,000/μL, 413 had 20–50,000/μL, 218 had 10–20,000/μL, and 85 had <10,000/μL [39]. The overall bleeding risk was 1.1%, while the risk of severe bleeding was 0.2%. Bleeding risk was not associated with platelet count. Severe bleeding included 2 cases of epistaxis, one in a patient with a central airway tumor and one in a patient with breast cancer and radiation-induced pneumonitis. Both non-nasal bleeding events occurred in patients with normal platelet count. The value of this study is significantly limited by the high proportion of patients who received platelet transfusions (90.6% of those with a platelet count < 10,000/μL and 55.5% of those with 10–20,000/μL), which was not adequately accounted for in the analysis. Additionally, 7.7% of patients were taking aspirin, and 10.8% were receiving anticoagulants. Another factor complicating the interpretation of the results is that in 92.4% of cases, bronchoscopy was performed via the nasal route.

In a second large retrospective study, bronchoscopy with BAL was performed in 507 patients with hematologic diseases and a platelet count < 50,000/μL, including 281 patients with very severe thrombocytopenia < 20,000/μL [40]. Platelet transfusions were administered in 81.5% of patients with platelet count < 20,000/μL and in 22.5% of those with platelet count > 20,000/μL. The risk of severe bleeding and hemoglobin decline was similar between the groups with a platelet count of 20–50,000/μL and <20,000/μL. The risk of non-severe bleeding was slightly higher in the <20,000/μL group (4.6% vs. 1.8%), but this difference was not statistically significant (*p* = 0.076). Clinically significant bleeding occurred in only 2 patients, both with platelet count < 20,000/μL. Multivariate analysis did not identify platelet count as a factor associated with bleeding risk.

In another single-center retrospective study involving 150 patients with platelet count < 100,000/μL, bronchoscopy (with BAL in some cases) was performed safely, with only 1 case of mild bleeding reported in a patient with a platelet count of 61,000/μL [41]. Prior to the procedure, 72 patients had platelet count < 50,000/μL and 15 had <20,000/μL. Platelet transfusions were administered to 65% of patients with platelet count < 50,000/μL and 8% of those with >50,000/μL. Several small studies focusing on specific patient populations were also identified. Kim et al. [42] performed bronchoscopy with BAL (*n* = 33) and TBLB (*n* = 5) in mechanically ventilated patients with platelet count < 50,000/μL, most of whom had hematologic malignancies. One episode of severe bleeding occurred, resulting in death within 24 h. In a prospective study of patients within 6 months following bone marrow transplantation, 66 bronchoscopies were performed in 47 patients with platelet count < 50,000/μL (including 13 with platelet count < 20,000/μL) [43]. There were 3 episodes of minor and self-limiting bleeding and 1 episode of severe epistaxis.

Insufficient data were available to draw conclusions about the safety of biopsy procedures. No studies were identified regarding EBB or TBLC. TBLB was reported in 5 patients in the study by Kim et al. [42] and in another study involving 22 patients with hematologic disorders [44]. In the latter study, biopsies were performed using a radial EBUS probe with a guide sheath. The mean platelet count was 12,000/μL (range: 7500–23,600/μL). No severe bleeding events were observed; however, all patients received platelet transfusions before or during the procedure. A conference abstract reported robot-assisted EBUS-TBNA in 35 patients with platelet count < 150,000/μL [45]. Two severe bleeding events were described in patients with platelet count < 50,000/μL, and an increased bleeding risk was noted in this subgroup compared to patients with platelet count > 50,000/μL.

#### 2.1.2. Information and Recommendations from Other Guidelines

The 2013 British Thoracic Society (BTS) guidelines state that bronchoscopy with BAL can be performed in patients with platelet count > 20,000 per μL, and that the local hematology team should be consulted regarding the need for platelet transfusion if EBB or TBLB is planned [6]. This is a grade D recommendation, the lowest level in the 4-tier system (A to D). The guidelines also recommended assessing platelet count when clinical risk factors suggest a likelihood of abnormal coagulation (also grade D). In addition, the evidence statement notes that “bleeding complications in patients with thrombocytopenia undergoing bronchoscopy and lavage are approximately 7%. No data are available regarding the safety of TBLB or EBB in thrombocytopenia but the majority of bleeding complications relate to epistaxis.” [6] (p. i10). The 2019 guidelines from the Indian Chest Society, National College of Chest Physicians, and Indian Association for Bronchology recommend a minimum platelet count of at least 20,000/μL for BAL and 50,000/μL for EBB or TBLB [7]. These are conditional recommendations based on low-quality evidence. The same document also states that “BAL can be performed in patients with platelet count <20,000 per mm^3^ if clinically indicated, after careful risk–benefit analysis. In patients with thrombocytopenia, oral route is preferred for performing bronchoscopy (UPP) [translator’s note: good practice point]” [7] (p. S47). According to the 2024 guidelines from the British Society of Haematology, platelet count alone should not be used as a predictor of bleeding risk. The decision to perform prophylactic platelet transfusion should take into account the cause of thrombocytopenia, platelet function, and the procedure-related risk, including both the patient’s condition and the nature of the intervention [17].

#### 2.1.3. **Recommendations**

- We suggest that the minimum platelet count required to safely perform bronchoscopy or bronchoscopy with BAL (without biopsy) should be ≥20,000/μL [conditional recommendation/low quality of evidence].
- For patients undergoing biopsy procedures (e.g., EBB, EBUS-TBNA, TBLB, TBLC), the platelet count should be ≥50,000/μL [conditional recommendation/very low quality of evidence].

#### 2.1.4. Commentary

These recommendations apply to cases of isolated thrombocytopenia, i.e., thrombocytopenia without coexisting hemostatic abnormalities that may increase bleeding risk. A platelet count ≥ 50,000/μL is generally considered sufficient for the safe performance of endoscopic procedures. A platelet count < 50,000/μL requires careful evaluation of the risk–benefit balance of bronchoscopy and the associated bleeding risk. In such cases, clinical decision-making should always consider the underlying cause of thrombocytopenia (e.g., immune thrombocytopenia, bone marrow suppression, bone marrow infiltration by malignancy, portal hypertension, or hypersplenism without splenomegaly). It is important to recognize that not all forms of thrombocytopenia are bleeding disorders—some represent prothrombotic states, such as heparin-induced thrombocytopenia, vaccine-induced immune thrombotic thrombocytopenia, thrombocytopenia in antiphospholipid syndrome, or thrombotic microangiopathies [46]. In some cases, laboratory results may reflect pseudothrombocytopenia caused by EDTA-dependent platelet clumping—this is an in vitro artifact that can lead to mismanagement. If suspected, repeat testing using blood collected in a heparin- or citrate-containing tube is recommended [47].

The recommendation of a platelet threshold of 20,000/μL for bronchoscopy without biopsy is not definitive (it is a conditional recommendation). The risk of significant bleeding also depends on several additional factors, including the endoscopist’s skill, the route of bronchoscope insertion (oral insertion is now preferred, as nasal insertion is associated with a higher risk of hemorrhagic complications), patient cooperation during the procedure, and other clinical variables. All of these factors should be carefully considered and weighed against the potential benefits of the procedure. When the expected clinical benefit outweighs the risks, bronchoscopy may be performed even in patients with a platelet count below 20,000/µL.

In patients with a platelet count below the recommended thresholds, it is advisable to consider postponing the procedure, implementing measures to increase the platelet count, and consulting a hematologist. Interventions aimed at increasing platelet count depend on the underlying cause of thrombocytopenia and most commonly include platelet transfusion, but may also involve pharmacologic treatments such as corticosteroids, intravenous immunoglobulins, thrombopoietin receptor agonists (romiplostim, eltrombopag, avatrombopag), and other immunosuppressive agents [48].

### 2.2. Question 2: In Patients with Abnormal INR and APTT, Is It Safe to Perform Bronchoscopy, BAL, EBB, EBUS-TBNA, TBLB, and TBLC? What Are the Maximum INR and APTT Values That Allow These Procedures to Be Performed Safely?

#### 2.2.1. Discussion of Scientific Evidence

Primary data: No primary studies directly addressing the clinical question were identified. However, several studies evaluated the predictive value of coagulation parameters (INR and APTT) for bleeding risk during bronchoscopy. In a prospective study evaluating 104 consecutive TBLB procedures performed in 51 patients (including some lung transplant recipients), clinically significant bleeding—defined as >20 mL of blood—occurred in 8 cases (7.7%). Bleeding risk did not correlate with coagulation parameters, although most patients likely had normal APTT and INR values [49].

In another retrospective study, 305 bronchoscopies were performed in 274 patients, of whom 28 had elevated APTT and/or INR (although the degree of abnormality was not reported). The incidence of bleeding in patients with abnormal coagulation parameters was not higher than in those with normal values [50]. A third study evaluated potential bleeding risk factors based on the volume of blood aspirated during bronchoscopy in 234 patients. It found that an INR > 1.3 was not associated with an increased risk of bleeding [51].

#### 2.2.2. Information and Recommendations from Other Guidelines

The 2019 guidelines of the Indian Chest Society, National College of Chest Physicians, and Indian Association for Bronchology do not recommend routine testing of coagulation parameters, platelet count, or hemoglobin levels prior to bronchoscopy (conditional recommendation) [7]. However, they suggest—as a good practice point—that such tests should be performed prior to bronchoscopy in patients with clinical risk factors for bleeding, including the use of anticoagulants, known bleeding disorders, or chronic liver or kidney disease. Similarly, the 2024 guidelines of the British Society of Haematology include a conditional recommendation advising against routine coagulation or platelet function testing to assess bleeding risk prior to invasive procedures [17].

#### 2.2.3. Recommendation: Due to Insufficient Evidence, No Formal Recommendation Has Been Made

**Good Practice Points**

- Available scientific data and clinical experience do not support the routine assessment of INR and APTT in patients undergoing bronchoscopy who are not receiving anticoagulant therapy, have no comorbidities warranting coagulation testing, and no clinical history suggestive of a bleeding tendency.
- Assessment of clinical bleeding risk factors is essential for evaluating the risk of hemorrhagic complications during bronchoscopy.

#### 2.2.4. Commentary

The authors of this guideline believe that current evidence and clinical practice do not justify routine INR and APTT testing in all patients undergoing evaluation for bronchoscopy. This applies to patients who: (1) do not have comorbid conditions associated with coagulopathy; (2) do not exhibit clinical signs suggestive of a bleeding disorder (e.g., a HEMSTOP score ≥ 2); and (3) are not taking anticoagulant drugs.

A laboratory test should be considered clinically useful if its result positively impacts diagnostic or therapeutic outcomes [52]. Current evidence indicates that routine coagulation screening tests—including APTT and PT, from which INR is calculated—do not meet this criterion of clinical usefulness in patients undergoing invasive procedures or surgery. In other words, estimating bleeding risk based solely on routine preprocedural APTT and INR testing lacks clinical justification, as prolonged APTT or elevated INR values do not correlate with an increased risk of periprocedural bleeding. More importantly, normal APTT and INR values do not exclude the presence of a bleeding disorder, such as von Willebrand disease, platelet function disorders, or mild clotting factor deficiencies.

Therefore, in patients without conditions known to significantly increase bleeding risk—such as advanced chronic liver or kidney disease, severe systemic infections, or other serious conditions that may cause coagulopathy (e.g., malnutrition or long-term antibiotic use)—and who are not receiving chronic anticoagulant therapy (e.g., VKAs) or DOACs, bleeding risk assessment should primarily rely on a structured personal and family history (e.g., using the HEMSTOP questionnaire [53]; Table 5), without simultaneous APTT or INR testing. If a patient scores < 2 points on the HEMSTOP questionnaire, invasive procedures, particularly those associated with a low risk of bleeding, can be performed without additional laboratory testing of plasma coagulation. The HEMSTOP questionnaire should not be viewed as a tool to quantify the bleeding risk before bronchoscopy (it was not validated in population of patients qualified for bronchoscopy) but rather as a tool to identify subjects who have increased bleeding risk and warrant additional investigation.

In all patients with signs or symptoms suggestive of a bleeding disorder and/or a HEMSTOP score ≥ 2, as well as in those with comorbidities that increase the risk of periprocedural bleeding, additional laboratory evaluation is warranted. In many cases, this extends beyond routine hemostasis screening tests. Therefore, consultation with a hematologist—preferably one specializing in bleeding and coagulation disorders—is recommended in such situations. Hematology consultation is also appropriate when abnormal hemostasis screening test results (e.g., prolonged APTT) are incidentally found in patients with a negative personal and family bleeding history, a low HEMSTOP score, and no significant comorbidities. This is important because some causes of prolonged APTT—such as the presence of lupus anticoagulant or deficiencies in factor XII, high-molecular-weight kininogen, or prekallikrein—are not associated with an increased risk of bleeding [54]. Appropriate guidance on further laboratory workup is necessary to promptly confirm such findings.

This practice point should not be viewed as a contraindication for performing coagulation tests in subjects qualified for diagnostic bronchoscopy, but rather as a suggestion that a clinical-based approach should be used here instead of relying solely on laboratory test results. When evaluating a patient for bronchoscopy, special attention should also be given to those with conditions known to increase the risk of bleeding during invasive procedures, particularly patients:

- With renal failure [55,56,57];
- With chronic liver disease, especially cirrhosis [58,59,60];
- Who are lung transplant recipients [61].

### 2.3. Question 3. In Patients Taking 1 or 2 Antiplatelet Agents, Is It Safe to Perform Bronchoscopy, BAL, EBB, EBUS-TBNA, TBLB, and TBLC?

#### 2.3.1. Discussion of Scientific Evidence

##### Patients Receiving Prophylactic-Dose Aspirin

Large observational studies, including high-quality prospective trials, have confirmed the safety of performing bronchoscopy with various additional procedures in patients receiving a prophylactic dose of aspirin. In a large prospective study excluding patients with coagulation disorders, Herth et al. [62] demonstrated the safety of TBLB. Lung biopsy was performed in 1217 patients, of whom 285 had taken aspirin within 24 h prior to the procedure. The rates of mild bleeding (1.8% in the aspirin group vs. 2.9% in the control group), moderate bleeding (1.1% vs. 1.4%), and severe bleeding (0.9% vs. 0.8%) were comparable and not statistically different between the groups. In another multicenter retrospective observational study evaluating bleeding risk during TBLC, 179 patients taking aspirin were compared with 845 controls. No significant differences were found between the groups in bleeding rates by severity [63]. In a univariate logistic regression analysis, aspirin use was not associated with an increased risk of clinically significant bleeding. Similar findings were reported in another retrospective cohort study involving 172 consecutive patients undergoing TBLC, 51 of whom were receiving aspirin [64]. No significant difference was found between the aspirin and control groups: any bleeding occurred in 60.8% vs. 63.6% of patients, respectively, and severe bleeding in 3.9% vs. 5%. Similarly, for EBUS-TBNA, no increased risk of bleeding was observed in patients taking aspirin [25,65]. Even in the setting of therapeutic bronchoscopy, including argon plasma coagulation and other interventions, aspirin use was not associated with an elevated bleeding risk [66].

It should be noted that data are limited for some procedures, particularly those considered less invasive [67]. However, evaluating individual bronchoscopic procedures separately may not be warranted in this context, as the available evidence supports the safe performance of even the highest-risk diagnostic bronchoscopic procedures, such as TBLB and TBLC, as well as surgery, including CABG, in patients receiving aspirin.

##### Patients Receiving Dual Antiplatelet Therapy

Dual antiplatelet therapy (DAPT), which combines a P2Y12 inhibitor with aspirin, is recommended for patients after percutaneous coronary intervention (PCI) or acute coronary syndrome (ACS) [68,69]. Many of these patients may require interventional procedures. Although specific data on the frequency of bronchoscopy in this population are lacking, observational studies suggest that the cumulative incidence of non-cardiac surgery after PCI is approximately 1% within 30 days, 5% within 6 months, and 9% within 1 year after the procedure [70,71]. Two low-quality cohort studies have evaluated the safety of performing EBUS in patients taking clopidogrel and aspirin. In a retrospective single-center analysis of 4859 patients undergoing EBUS-TBNA, 102 had either delayed discontinuation of antiplatelet therapy or did not discontinue it at all. Among them, 86 were receiving both aspirin and clopidogrel (13 were taking cilostazol, and 45 were taking aspirin). No increased incidence of bleeding was observed in the group with delayed drug discontinuation [72]. Similar findings were reported in a smaller retrospective study involving 295 patients, of whom 42 were taking aspirin and clopidogrel [73]. However, in another retrospective analysis of 406 patients undergoing EBUS-TBNA, 23 were on dual therapy, 99 were on aspirin alone, 13 were on clopidogrel alone, and 271 served as controls [25]. In contrast to the previously mentioned study, bleeding occurred more frequently in the DAPT group compared to both the aspirin-only and control groups (8.7% vs. 0% vs. 2.6%). Patients receiving DAPT also had a greater risk of hemoglobin decline. Additionally, several case series have reported the safe performance of EBUS-TBNA or EBB in patients taking both aspirin and clopidogrel, or clopidogrel alone [74,75,76]. Importantly, discontinuation of the second antiplatelet agent has been associated with an increased risk of adverse cardiovascular events. This was highlighted in a large retrospective analysis of 1489 patients undergoing EBUS-TBNA, in which only 16 had not discontinued clopidogrel or prasugrel within 5 days of the procedure. The risk of mild bleeding in this group was significantly higher (25% vs. 5.4% in the control group); however, no severe bleeding events were reported. In contrast, among patients who discontinued antiplatelet therapy, 2 experienced non-ST-segment-elevation myocardial infarction after the procedure, resulting in death in 1 case [77].

A large prospective study reported a markedly increased risk of bleeding during TBLB in patients taking clopidogrel, either alone (18 patients) or in combination with aspirin (12 patients) [3]. The study was terminated early due to the high rate of bleeding in the clopidogrel group vs. controls: mild bleeding occurred in 27% vs. 1.5%, moderate bleeding in 34% vs. 1.5%, and severe bleeding in 27% vs. 0.3% (all *p* < 0.001). Among patients receiving both clopidogrel and aspirin, bleeding occurred in 100% of cases: 50% experienced moderate bleeding and 50% severe [3]. Clopidogrel use was also associated with a significantly increased bleeding risk during TBLC [64]. In a retrospective study of 172 consecutive patients undergoing lung cryobiopsy, the use of clopidogrel in combination with aspirin was associated with a significantly elevated bleeding risk (odds ratio, 9.8).

Regarding cardiovascular risks associated with modification of DAPT in patients after PCI, observational studies have demonstrated an increased incidence of adverse cardiovascular events, including cardiac death, myocardial infarction, and stent thrombosis, following surgical procedures. The estimated risk ranges from 2% to 8% [78,79,80], representing more than a twofold increase compared to patients without coronary stents. Stent thrombosis is associated with worse prognosis than de novo coronary artery occlusion.

#### 2.3.2. Information and Recommendations from Other Guidelines

The 2013 BTS guidelines recommend discontinuing clopidogrel 7 days prior to considering EBB or TBLB, while low-dose aspirin alone may be continued [6]. This is a grade C recommendation. The 2019 guidelines of the Indian Chest Society, National College of Chest Physicians, and Indian Association for Bronchology include the following recommendations:

(1) Clopidogrel, prasugrel, or ticagrelor should be discontinued at least 5 days before EBB and TBLB (grade 2A).
(2) Consultation with an appropriate specialist is recommended to modify antiplatelet therapy in patients on DAPT and at high risk of thrombosis (good practice point) [7].

Periprocedural management of patients receiving antiplatelet therapy is also addressed in the 2022 guidelines of the American College of Chest Physicians (ACCP) [18]. In reference to invasive procedures performed during antiplatelet therapy, the authors suggest the following:

(1) In patients taking aspirin undergoing elective non-cardiac surgery, aspirin should be continued rather than stopped (conditional recommendation, moderate-quality evidence).
(2) In patients taking aspirin and a P2Y12 inhibitor who have undergone coronary stent placement within the past 3–12 months and are scheduled for elective surgery/invasive procedures, the P2Y12 inhibitor should be stopped before surgery rather than continued (conditional recommendation, very low-quality evidence).
(3) In patients with coronary stents who require interruption of antiplatelet therapy for elective surgery or invasive procedures, routine bridging with glycoprotein IIb/IIIa inhibitors, cangrelor, or LMWH should not be used (conditional recommendation, low-quality evidence).

#### 2.3.3. **Recommendations**

- In patients receiving low-dose aspirin, discontinuation of the drug is not required before bronchoscopy, BAL, EBB, EBUS-TBNA, TBLB, or TBLC [strong recommendation, moderate quality of evidence].
- In patients requiring DAPT, defined as a prophylactic dose of aspirin combined with a P2Y12 receptor inhibitor (clopidogrel, prasugrel, or ticagrelor), an individualized assessment is recommended. This should weigh the diagnostic benefit of bronchoscopy against the bleeding risk associated with the procedure and the risk of stent thrombosis resulting from antiplatelet discontinuation [conditional recommendation, very low quality of evidence].

#### 2.3.4. Commentary

Low-dose aspirin is defined as ≤150 mg. There is moderate-quality evidence indicating that performing bronchoscopic procedures while continuing prophylactic-dose aspirin does not increase the risk of bleeding [62,63,64,65]. Even TBLC, the diagnostic procedure associated with the highest bleeding risk, can be performed safely in patients taking low-dose aspirin. In addition, evidence from other surgical settings, such as coronary artery bypass grafting, supports the safety of continuing aspirin, and this approach is endorsed by major scientific societies [18].

**Figure 1:**
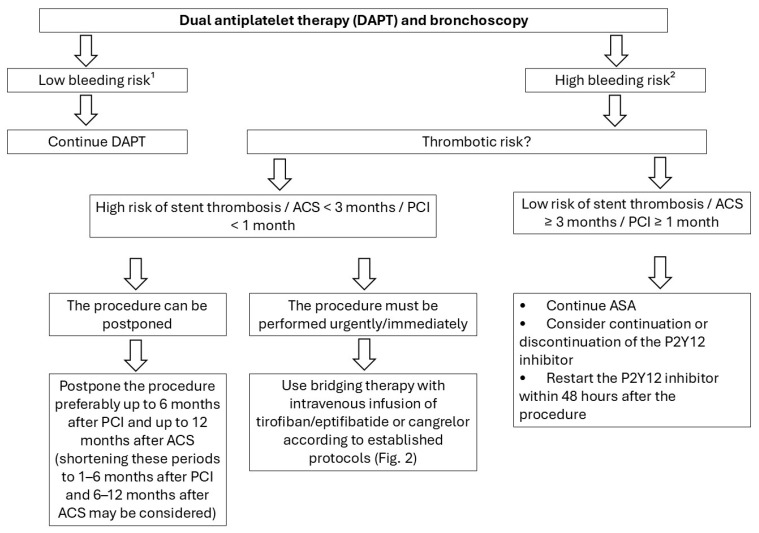
Suggested management in patients receiving DAPT (based on the 2022 European Society of Cardiology guidelines [84], modified). ^1^ Bronchoscopy without additional examinations, ^2^ Bronchoscopy with biopsy procedures.

**Figure 2:**
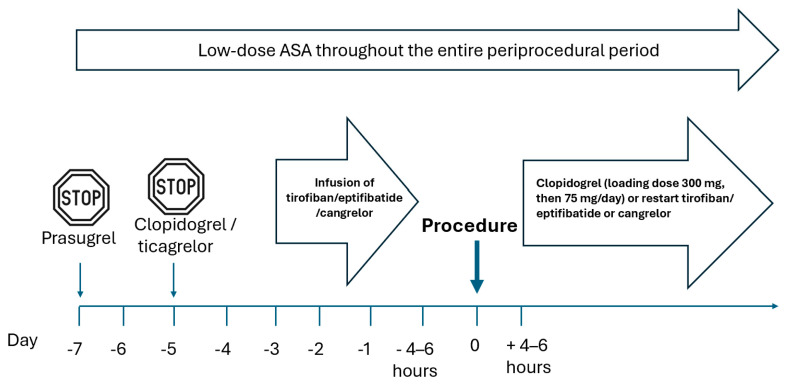
Principles of bridging therapy in patients receiving DAPT (based on the 2022 European Society of Cardiology guidelines [84], modified).

In patients receiving DAPT, the decision to proceed with diagnostic bronchoscopy cannot be reduced to a procedural risk–benefit calculation; it must also account for the cardiovascular consequences of antiplatelet interruption. The clinical yield of bronchoscopy and the bleeding risk it carries are necessary considerations, but they are not sufficient—periprocedural cardiovascular risk constitutes a third, coequal axis that determines whether, and how, DAPT should be modified. This trade-off is addressed in detail below; in all cases, decisions should be anchored to current cardiology guidance, including the 2022 ESC guidelines. DAPT increases the risk of bleeding during lung biopsy procedures. Although data regarding EBUS-TBNA are inconsistent and of low quality, some studies have reported an increased bleeding risk in patients receiving DAPT [3,64]. Therefore, it appears reasonable to recommend avoiding biopsy procedures in patients on DAPT. However, there is also evidence indicating that discontinuation of the second antiplatelet agent (i.e., the P2Y12 inhibitor) in many clinical scenarios increases the risk of ischemic events [81,82,83]. Thus, the decision to withhold antiplatelet therapy should be made with caution. In such patients, a thorough interdisciplinary evaluation is essential to assess the balance between the bleeding risk associated with the planned procedure and the risk of myocardial ischemia. Based on this assessment, appropriate management strategies may include proceeding with bronchoscopy while continuing DAPT, postponing the procedure, temporarily withholding the P2Y12 inhibitor, or implementing bridging therapy (Figure 1 and Figure 2). The latter option is not widely available and is currently used infrequently. Bridging therapy should be reserved for patients who meet the following criteria:

(1) Urgent indication for bronchoscopy;
(2) Increased bleeding risk if the procedure is performed while DAPT is continued (e.g., in the context of a planned high-bleeding-risk procedure such as transbronchial lung cryobiopsy, TBLC);
(3) Increased risk of stent thrombosis (Table 6);
(4) Potential harm associated with postponing the procedure (e.g., delayed diagnosis of potentially treatable lung cancer).

**Table 6:** Clinical scenarios associated with high risk of stent thrombosis in the periprocedural period (adapted from [69]).

History of stent thrombosis while on antiplatelet therapy
Reduced left ventricular contractility (left ventricular ejection fraction < 40%)
Uncontrolled diabetes mellitus
Severe renal impairment (commonly defined as estimated glomerular filtration rate [eGFR] < 30 mL/min)
Poor stent apposition or dissection observed during PCI or on follow-up coronary angiography
Other procedural factors deemed high risk by the invasive cardiologist, such as complex PCI, PCI involving heavily calcified lesions, left main coronary artery stenosis, chronic total occlusion, bifurcation lesions/use of the crush technique, and PCI of bypass grafts.

If discontinuation of the P2Y12 inhibitor is necessary, ticagrelor should be stopped 3–5 days before the procedure, clopidogrel 5 days before, and prasugrel 7 days before. The procedure should be performed without discontinuing aspirin.

The preferred strategy after PCI is to delay any planned diagnostic procedure until completion of the full course of DAPT, that is, 6 months following elective PCI and 12 months after ACS. If this is not feasible, bronchoscopy should be postponed at least until completion of the first month of DAPT. In patients at high cardiovascular risk (e.g., following ACS), continuing DAPT for at least 3 months before withdrawal should be considered. If the P2Y12 inhibitor is discontinued, the procedure should be performed while aspirin is continued.

In cases where DAPT must be interrupted (i.e., discontinuation of the P2Y12 receptor inhibitor) before bronchoscopy, it is suggested that diagnostic or therapeutic procedures be performed in facilities with 24-h access to a catheterization laboratory for patients:

- Within 6 months of PCI without recent ACS and without high ischemic risk;
- Within 12 months of recent ACS or in those at high ischemic risk.

The antiplatelet management strategy in patients with a recent history of PCI who require bronchoscopy should be determined through collaborative decision-making between the pulmonologist and the cardiologist. Bridging therapy with intravenous antiplatelet agents (such as eptifibatide, tirofiban, or cangrelor) is generally not recommended. However, it may be considered in exceptional cases, but only if both of the following conditions are met:

- Postponing bronchoscopy would pose a significant health risk to the patient;
- Discontinuation of DAPT prior to the pulmonary procedure is not feasible (e.g., in patients at very high risk of stent thrombosis, with a history of recurrent myocardial infarction, or recent PCI).

### 2.4. Question 4. In Patients Taking Oral Anticoagulants, Is It Safe to Perform Bronchoscopy, BAL, EBB, EBUS-TBNA, TBLB, or TBLC? If Not, When Should Anticoagulation Be Discontinued Before the Procedure, and When Should It Be Resumed Afterward?

#### 2.4.1. Discussion of Scientific Evidence

The identified studies are limited to small sample sizes, insufficient adjustment for confounding factors, or a lack of direct relevance to the clinical question. In the only prospective study identified, which was conducted in a small cohort, bronchoscopy with BAL, brush biopsy, and TBLB were performed in 4 patients taking warfarin and 23 control patients. The volume of blood loss during the procedure was comparable between groups, and no severe bleeding events were reported [85]. In a large retrospective cohort study, the safety of bronchoscopy was evaluated in 603 patients receiving DOACs compared with 2320 matched controls not taking these agents (controls matched 4:1 to the DOAC group based on treatment center, sex, age, and year of procedure) [86]. The overall risk of bleeding was slightly higher in the DOAC group compared to the controls (1.3% vs. 0.8%); however, bleeding risk was not assessed using multivariate analysis. Multivariate analysis was performed only for a composite endpoint that included bleeding, along with mechanical ventilation, pneumothorax, and thromboembolism, and this composite endpoint occurred more frequently in the DOAC group. However, detailed information regarding the types of bronchoscopic procedures performed was not reported, and the study population had distinctive features, such as advanced age, multiple comorbidities, and a markedly high risk of in-hospital mortality.

In another publication, a retrospective analysis was conducted in patients who discontinued DOACs ≤ 96 h before the procedure (with timing based on the specific drug and creatinine clearance, typically ranging from 48 to 96 h). In this cohort, 7 EBUS-TBNA procedures, 1 EBUS without TBNA, 2 endobronchial biopsies, and 7 diagnostic bronchoscopies were performed. No severe bleeding events were reported; however, 3 cases of mild bleeding and 1 clinically relevant non-major bleeding were described [87]. No difference in bleeding risk was found between patients with drug concentrations < 30 ng/mL and those with >30 ng/mL. In a retrospective cohort study of 4271 patients undergoing EBUS-TBNA, 498 were identified as receiving antiplatelet and/or anticoagulant therapy. Among them, 5 patients taking DOACs had not discontinued the medication within the recommended timeframe [72]. Performing EBUS-TBNA in these patients was not associated with increased bleeding risk. A 2019 meta-analysis found no studies involving patients on oral anticoagulants undergoing bronchoscopy [67]. A 2022 retrospective analysis evaluated 820 patients undergoing various procedures, including gastroscopy, interventional cardiac procedures, or minor dermatologic interventions. In approximately 45% of patients, DOACs were temporarily discontinued, which was associated with a lower risk of bleeding [88]. Available review articles generally suggest stopping VKAs 5 days prior to the procedure, verifying the INR on the day of the procedure, and resuming VKAs 24 h afterward [89,90,91]. For DOACs, the suggested discontinuation interval depends on the specific agent and renal function, ranging from 1 to 4 days [92,93,94,95]. In one study assessing gastrointestinal endoscopy procedures, the mean DOAC discontinuation period was 3.9 days [96]. For minor procedures with low risk of severe bleeding (<2%), some sources suggest either continuing anticoagulation or withholding DOACs only on the day of the procedure [93,97,98].

#### 2.4.2. Information and Recommendations from Other Guidelines

In the 2013 BTS guidelines, it is suggested only that “anticoagulants should be managed according to published guidelines …” [6] (p. i10). The 2019 guidelines of the Indian Chest Society, National College of Chest Physicians, and Indian Association for Bronchology state that DOACs should be discontinued at least 2 days before TBNA or bronchoscopic biopsy (3A). Bridging therapy with LMWH is recommended in patients on anticoagulation who are at high risk of thrombosis, while BAL can be performed in patients on anticoagulants after careful risk–benefit analysis, preferably during bronchoscopy performed via the oral route (good practice point) [7].

The 2024 British Society for Haematology guidelines on bleeding risk assessment before nonsurgical invasive procedures do not provide formal recommendations specific to bronchoscopy. However, the document suggests discontinuing DOACs 2 days before procedures (except for those with low bleeding risk) and discontinuing VKAs 5 days before the procedure [17].

The current Australian Clinical Excellence Commission guidelines from 2025 suggest performing low-bleeding-risk procedures while patients are on VKAs, provided that the INR is within the therapeutic range. For procedures with low to medium bleeding risk, including bronchoscopy with or without biopsy, temporary interruption of VKAs is advised [99].

The 2022 ESC guidelines on cardiovascular risk assessment and management in patients undergoing non-cardiac surgery provide several useful algorithms for managing patients on VKAs and DOACs [84]. Among other recommendations, the guidelines state that for interventions associated with a very high risk of bleeding—such as central neuraxial blocks (spinal and/or epidural anesthesia) and lumbar puncture—which require normal hemostasis, consideration should be given to withholding DOACs for up to 5 half-lives of the drug (i.e., approximately 3 days for factor Xa inhibitors and 4–5 days for dabigatran). Resumption of DOAC therapy is typically possible 24 h after the intervention [84].

The American College of Chest Physicians (ACCP) guidelines also provide detailed recommendations on the periprocedural management of VKAs and DOACs [18]. Examples include not interrupting VKA therapy before pacemaker implantation and not using heparin bridging in patients whose VKA therapy is discontinued for colonoscopy with probable polypectomy. The ACCP document also provides guidance on the specific timing of anticoagulant discontinuation for various agents.

#### 2.4.3. Recommendation: Due to Insufficient Direct Evidence, No Formal Recommendation Has Been Made

**Good Practice Points**

- Although no high-quality data specifically address bronchoscopic procedures in patients receiving oral anticoagulants, numerous guidelines provide recommendations for periprocedural management in this population. Adherence to these guidelines is advised.

In patients currently receiving oral anticoagulants:

- Bronchoscopy or bronchoscopy with BAL is acceptable.
- For patients taking DOACs, the procedure should ideally be scheduled at the drug’s trough concentration (typically 12 or 24 h after the last dose).
- For patients receiving VKAs, INR measurement before the procedure is recommended.
- Biopsy procedures should generally be avoided.

If a decision is made to temporarily interrupt anticoagulant therapy:

- The recommended timing for discontinuation of DOACs before bronchoscopy with planned biopsy procedures is outlined in Table 7 of the full guideline text.
- VKAs should be discontinued 5 days before the procedure, and the INR should be checked on the day of the procedure.
- Routine periprocedural bridging with heparin is not recommended in patients whose oral anticoagulant therapy was discontinued before the procedure, except in selected patients at very high risk of thromboembolic events (Table 8).

#### 2.4.4. Commentary

It is estimated that 1 in 4 patients receiving anticoagulant therapy may require interventional treatment within 2 years of initiation of treatment [101]. The literature review revealed relatively limited data on bronchoscopic procedures in patients taking oral anticoagulants, likely due to the long-standing periprocedural management practices already established for such patients undergoing interventional procedures. Due to the lack of high-quality, bronchoscopy-specific evidence, no formal recommendations could be formulated. However, to support users of these guidelines, good practice points have been developed, based on existing perioperative management recommendations for VKAs and DOACs, as well as the clinical expertise of the panel members.

**Figure 3:**
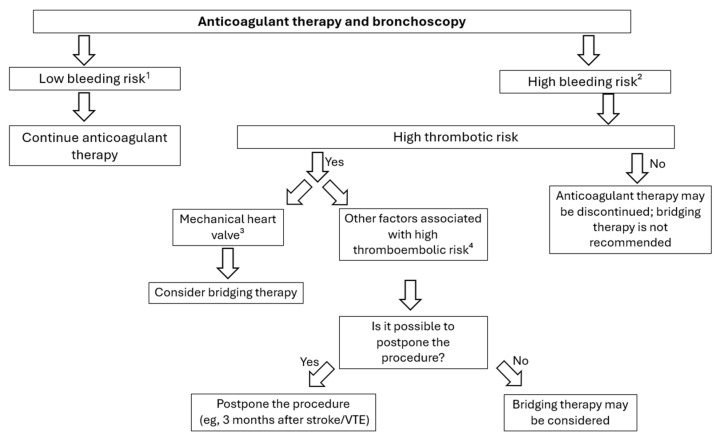
Periprocedural management algorithm for patients on oral anticoagulants (based on the ESC 2022 guidelines [84], modified). ^1^ Bronchoscopy without additional interventions. ^2^ Bronchoscopy with biopsy procedures. ^3^ Mechanical heart valve in the aortic position with any thromboembolic risk factor (e.g., atrial fibrillation, prior thromboembolic event, severe left ventricular dysfunction, hypercoagulable state); older-generation mechanical aortic valve; mechanical valve in the mitral position. ^4^ Stroke or venous thromboembolism (VTE) within the past 3 months; high risk of recurrent VTE (e.g., antithrombin III, protein C and/or S deficiency, antiphospholipid syndrome); left ventricular apical thrombus; atrial fibrillation with very high stroke risk (CHA_2_DS_2_-VASc score > 6 points).

The modification of oral anticoagulant therapy in the periprocedural period should be guided by factors related to both the extent of planned bronchoscopy and the patient’s clinical status, as well as the type of oral anticoagulant used (VKA or DOAC) (Figure 3). In most patients, DOACs are preferred due to their lower risk of bleeding complications and greater convenience for patients [102]. Currently, three DOAC agents are available in Poland: dabigatran (a factor IIa inhibitor), as well as apixaban and rivaroxaban (factor Xa inhibitors).

Direct oral anticoagulants (DOACs) have a predictable offset of anticoagulant effect; therefore, withholding them for a defined period (Table 7) is generally considered safe for most patients. In situations where DOAC accumulation may occur (e.g., renal impairment, older age, or interactions with drugs such as amiodarone, digoxin, diltiazem, clarithromycin, or fluconazole), the discontinuation period should be extended by 12–24 h beyond the standard time.

When temporary discontinuation of DOACs is required prior to a procedure, routine use of bridging therapy is not recommended. An exception may be made for a small subset of patients at very high thromboembolic risk, but such decisions should be made individually, weighing the risk of bleeding versus thromboembolism.

In patients with mechanical heart valves (MHV) receiving treatment with VKAs, maintaining a therapeutic INR is essential to prevent thromboembolic complications. When bronchoscopy with biopsy is planned—requiring INR ≤ 1.5—interruption of VKA therapy is necessary, and bridging with heparin should be considered. In all cases, a risk–benefit assessment is required, balancing the bleeding risk against the benefits of thromboembolism prevention.

In patients with MHV at high thromboembolic risk (Table 8), bridging anticoagulation should be considered during the perioperative period when the INR falls outside the therapeutic range [84,102]. In patients with atrial fibrillation or venous thromboembolism, bridging therapy may be considered for those deemed at high risk of thromboembolic events (Table 8). However, all decisions regarding bridging should be based on an individual assessment of bleeding versus thromboembolic risk.

Unfractionated heparin (UFH), administered intravenously, remains the only heparin preparation officially approved for bridging therapy in patients with MHVs. However, in clinical practice, LMWHs, administered subcutaneously, are increasingly used off-label as a substitute for UFH in this setting. This trend reflects LMWH’s more favorable safety profile (e.g., lower risk of heparin-induced thrombocytopenia), ease of administration, more predictable pharmacokinetics and pharmacodynamics, and reduced treatment costs (facilitating outpatient bridging therapy). When using LMWH, therapeutic dosing twice daily is recommended, with dose adjustments as needed based on renal function [103,104,105].

In patients whose VKA therapy was interrupted prior to the procedure, treatment should be resumed within 12–24 h after the procedure, provided that hemostasis has been achieved. It is initially recommended to increase the maintenance VKA dose by 50% for the first 2 days. For patients who received bridging therapy, resumption of VKA treatment should be accompanied by concurrent administration of LMWH or UFH, with heparin continued until a therapeutic INR level is achieved.

DOAC therapy may be restarted 6–8 h after the procedure, provided effective hemostasis has been achieved. However, using reduced DOAC solely to mitigate bleeding risk is not recommended, as there is no scientific evidence supporting its efficacy or safety.

### 2.5. Question 5. Is It Safe to Perform Bronchoscopy, BAL, EBB, EBUS-TBNA, TBLB, and TBLC in Patients Receiving Prophylactic or Therapeutic Low-Molecular-Weight Heparin (LMWH), and if Not, When Should LMWH Be Withheld Before the Procedure and When Should It Be Restarted Afterward?

#### 2.5.1. Discussion of Scientific Evidence

No primary studies were identified that directly address this clinical question. Several primary studies have reported on various endoscopic procedures, including highly invasive ones, performed in patients treated with heparin. However, these were descriptive studies without control groups, which limits the ability to draw conclusions about relative bleeding risk. In one case series, percutaneous tracheostomy with assistance from rigid bronchoscopy was safely performed in patients receiving LMWH. Notably, in 26.9% of cases, the procedure was carried out while a therapeutic dose of heparin was still being administered [106]. Additionally, a review article summarized 48 publications, encompassing 107 cases in which various procedures were performed during extracorporeal membrane oxygenation (ECMO) with concurrent heparin use. These included multiple bronchoscopies involving stent placement and the management of endobronchial tumors. However, only aggregate data are available, indicating that airway bleeding occurred in 3.9% of patients [107]. Another report described the experience of a single center, which included 14 rigid bronchoscopies with stent implantation, exchange, or removal during ECMO; 4 bleeding events were noted, one of which required blood transfusion [108]. It should be noted that these studies primarily involved more invasive procedures than the diagnostic bronchoscopic procedures addressed in the current guidelines. A systematic review found no studies specifically addressing bronchoscopy during LMWH therapy [67].

The question of whether LMWH should be withheld before bronchoscopy has been discussed in numerous review articles. In the 2017 study by Abuqayyas et al. [89], it is recommended to discontinue LMWH 24 h before bronchoscopy if dosed at 2 × 1 mg/kg body weight per day, or 36 h before the procedure if dosed at 1 × 1.5 mg/kg body weight per day. The study suggests resuming LMWH 24–72 h after the procedure. The suggestion to discontinue LMWH 24 h before pulmonary procedures is also presented in the 2017 review article by Pathak et al. [109]. Another review article by Bernasconi et al. [19] stated that bronchoscopy or BAL can be performed while on LMWH therapy; however, for procedures involving biopsy, the approach depends on the LMWH dosing regimen:

(1) Prophylactic dosing: LMWH should be discontinued 10–12 h before the procedure and resumed on the same day.
(2) Therapeutic dosing: LMWH should be discontinued 24 h before the procedure and resumed 4–12 h afterward [19].

#### 2.5.2. Information and Recommendations from Other Guidelines

The 2013 BTS guidelines do not provide recommendations on this topic [6]. The 2019 guidelines issued by the Indian Chest Society, National College of Chest Physicians, and Indian Association for Bronchology recommended administering the last dose of LMWH 24 h prior to the procedure. However, this recommendation pertains specifically to bridging therapy in the context of interrupted oral anticoagulant therapy, and it is unclear whether the authors intended it to apply to discontinuation of LMWH therapy itself [7]. Several other documents outline periprocedural management strategies for patients receiving heparin. The Thrombosis Canada clinical practice guidelines state, among other points, that there is generally no indication to discontinue prophylactic LMWH before most procedures. For once-daily prophylactic dosing, the Thrombosis Canada guidelines suggest administering the last dose 2 days before the procedure. For selected patients receiving twice-daily dosing, the last dose may be given more than 24 h before the intervention [110]. In contrast, the Scottish National Health Service guidelines advise administering the final prophylactic dose of LMWH 12 h before the procedure, and for therapeutic dosing—24 h prior, provided the patient has normal renal function [111]. The ACCP guidelines, in the context of bridging therapy with heparin, also address LMWH discontinuation. They suggest—based on a conditional recommendation and very low-quality evidence—that the last therapeutic dose of LMWH be given approximately 24 h before the procedure [18].

#### 2.5.3. Recommendation: Due to Insufficient Direct Evidence, No Formal Recommendation Has Been Made

**Good practice points:**

- For bronchoscopy with biopsy, it is suggested to discontinue LMWH prior to the procedure as follows: 12 h for prophylactic dosing and 24 h for therapeutic dosing.
- For bronchoscopy without additional examinations, the procedure can be performed while continuing prophylactic LMWH.
- Resumption of LMWH after the procedure: If no bleeding complications occurred during the procedure, or if adequate hemostasis was achieved, LMWH may be resumed ≥8 h after the procedure.

#### 2.5.4. Commentary

**Figure 4:**
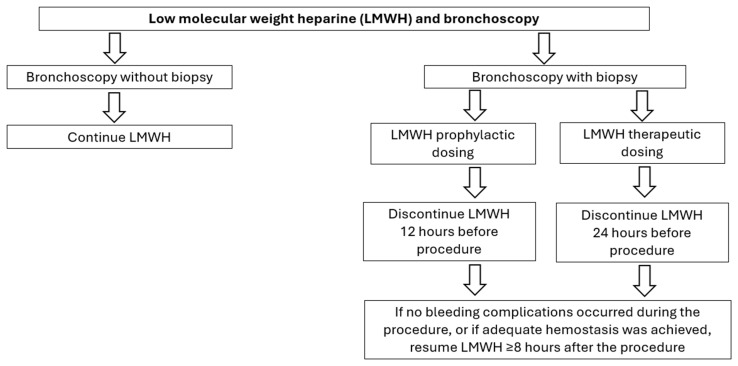
Periprocedural management algorithm for patients on LMWH.

An algorithm for periprocedural LMWH management is presented in Figure 4. Due to its predictable pharmacologic effects, LMWH is typically administered without routine laboratory monitoring. As a result, in most patients, routine discontinuation of LMWH prior to procedures is feasible within a similar time frame. However, given the lack of high-quality bronchoscopy-specific evidence, no formal recommendation has been formulated for this clinical question. Based on existing guidelines and clinical practice statements regarding the discontinuation of LMWH before invasive procedures, as well as data on non-bronchoscopic interventions and the authors’ clinical experience, good practice points have been formulated. These should be regarded as general guidance only. Each case should be assessed individually, taking into account the diagnostic benefits of bronchoscopy, the bleeding risk associated with the procedure, and the potential consequences of interrupting LMWH therapy.

If BAL is planned while LMWH is being administered, it is preferable to schedule the procedure during the drug’s trough period (just before the next dose) to minimize the risk of blood contamination in the sample and any potential reduction in its diagnostic value.

## 3. Guidelines Implementation and Update Plans

### 3.1. Guidelines Implementation Plan

The document will be submitted for publication in English in a peer-reviewed journal and then published in Polish on the PTChP website. The guidelines will also be discussed at the upcoming PTChP meeting and presented at PTChP-hosted training sessions.

### 3.2. Guidelines Update Plan

A supplementary literature review is scheduled to be conducted two years after the guidelines’ publication date. Based on the results of this review, the Chair of the Working Group will decide whether the document requires updating.

## Figures and Tables

**Table 1:** Bleeding severity scale used in the development of the guideline and corresponding outcome weights [2,3,4,5].

Bleeding Severity	Definition	Outcome Weight (Scale 1–9)
None	No bleeding	n/a
Mild	Bleeding controlled with suction alone	Not important (1/9)
Moderate	Bleeding requiring temporary bronchial occlusion with the bronchoscope or endobronchial administration of cold saline, adrenaline, or thrombin	Important but not critical (4/9)
Severe	Bleeding requiring more advanced interventions such as bronchial blocker placement, thoracic surgery, or blood transfusion; or that associated with clinically significant hemodynamic instability, severe respiratory failure, need for resuscitation, intensive care unit admission, or death	Critical (9/9)

**Table 2:** Classification of recommendation strength and its interpretation according to GRADE (based on [1]).

Strength of Recommendation	Interpretation		
	Clinicians	Patients	Health Policy Makers
Strong	Most individuals should receive the recommended course of action. Adherence to this recommendation according to the guideline could be used as a quality criterion or performance indicator. Formal decision aids are not likely to be needed to help individuals make decisions consistent with their values and preferences.	Most individuals in this situation would want the recommended course of action and only a small proportion would not.	The recommendation can be adapted as policy in most situations including for the use as performance indicators.
Weak (conditional)	Clinicians should recognize that different choices will be appropriate for different patients, and that they must help each patient arrive at a management decision consistent with her or his values and preferences. Decision aids may well be useful helping individuals make decisions consistent with their values and preferences. Clinicians should expect to spend more time with patients when working towards a decision.	The majority of individuals in this situation would want the suggested course of action, but many would not.	Policy-making will require substantial debates and the involvement of many stakeholders. Policies are also more likely to vary between regions. Performance indicators would have to focus on the fact that adequate deliberation about the management options has taken place.

**Table 3:** Classification of quality of evidence according to GRADE (based on [1]).

Quality of Evidence Grades	Definition
High	We are very confident that the true effect lies close to that of the estimate of the effect.
Moderate	We are moderately confident in the effect estimate: The true effect is likely to be close to the estimate of the effect, but there is a possibility that it is substantially different.
Low	Our confidence in the effect estimate is limited: The true effect may be substantially different from the estimate of the effect.
Very low	We have very little confidence in the effect estimate: The true effect is likely to be substantially different from the estimate of effect.

**Table 4:** Estimated risk of severe bleeding during the most common bronchoscopic procedures [4,22,23,24,25,26,27,28,29,30,31,32,33,34,35,36,37,38].

Procedure	Bleeding Risk	Risk of Severe Bleeding
Diagnostic bronchoscopy without additional interventions	<0.12–0.38%	Extremely rare
BAL	0.17%	0.004%
EBUS-TBNA	0.2–2.9%	<0.01%
EBB	0.12–17.2%	<0.1%
TBLB	1.9–5% *	<0.1–4% *
TBLC	15–30% *	<0.1−13.1% *

* Reported bleeding rates for transbronchial lung biopsy, particularly TBLC, vary considerably across the available literature. For example, some studies have reported bleeding of any severity in up to 72.9% of patients undergoing TBLC and 48.2% of those undergoing TBLB.

**Table 5:** HEMSTOP questionnaire.

HEMSTOP Questionnaire	
Have you ever consulted a doctor or received treatment for prolonged or unusual bleeding (such as nosebleeds, minor wounds)?	yes no
Do you experience bruises/hematomas larger than 2 cm without trauma or severe bruising after minor trauma?	yes no
After a tooth extraction, have you ever experienced prolonged bleeding requiring medical/dental consultation?	yes no
Have you experienced excessive bleeding during or after surgery?	yes no
Is there anyone in your family who suffers from a coagulation disease (such as hemophilia, von Willebrand disease, etc.)?	yes no
**For females:**	
Have you ever consulted a doctor or received treatment for heavy or prolonged menstrual periods (contraceptive pill, iron, etc.)?	yes no
Did you experience prolonged or excessive bleeding after delivery?	yes no
**Two or more positive answers suggest increased bleeding risk.**	

Based on Bonhomme et al. [53], published with the author’s permission.

**Table 7:** Proposed timing for DOAC discontinuation before bronchoscopy (based on [84,92,93,94], modified).

Renal Function (eGFR mL/min)	Dabigatran		Apixaban/Rivaroxaban	
	Low Bleeding Risk ^1^	High Bleeding Risk ^2^	Low Bleeding Risk ^1^	High Bleeding Risk ^2^
≥80	≥24 h	≥48 h	≥24 h	≥48 h
50–79	≥36 h	≥72 h		
30–49	≥48 h	≥96 h		
15–29	Not recommended		≥36 h	
<15	No formal indications for use			

^1^ BAL, EBUS-TBNA, ^2^ EBB, TBLB, TBLC.

**Table 8:** Clinical scenarios associated with a high risk of thromboembolic complications (based on [84,100], modified).

Anticoagulation	Clinical Scenario
VKA due to mechanical heart valve	Mechanical valve in the aortic position plus any thromboembolic risk factor (e.g., atrial fibrillation, prior thromboembolic event, severe left ventricular dysfunction, hypercoagulable state)
	Older-generation mechanical heart valve in the aortic position
	Mechanical heart valve in the mitral position
Other indications for VKAs or prophylactic use of DOACs	Recent ischemic stroke or VTE (<3 months)
	High risk of VTE recurrence (e.g., antithrombin III, protein C and/or S deficiency, antiphospholipid syndrome)
	Left ventricular apical thrombus
	Atrial fibrillation with very high risk of stroke (CHA_2_DS_2_-VASc > 6 points) Stroke, TIA, or peripheral embolism within the last 3 months
	Prior thromboembolic event during previous interruption of DOAC therapy (applies to DOAC-treated patients only)

## Data Availability

No new data were created or analyzed in this study. Data sharing is not applicable to this article.

## Supplementary Materials

The following supporting information can be downloaded at: https://www.mdpi.com/article/10.3390/arm94040044/s1, Supplementary Material File S1: detailed clinical questions; Supplementary Material File S2. Literature search; Figure S1: Flowchart illustrating the selection process of reports.

## Abbreviations

The following abbreviations are used in this manuscript:

ACS	Acute coronary syndrome
APTT	Activated partial thromboplastin time
BAL	Bronchoalveolar lavage
DAPT	Dual antiplatelet therapy
DOAC	Direct oral anticoagulants
EBB	Endobronchial biopsy
EBM	Evidence-based medicine
EBUS-TBNA	Transbronchial needle aspiration performed under endobronchial ultrasound guidance
ECMO	Extracorporeal membrane oxygenation
EDTA	Ethylenediaminetetraacetic acid
GRADE	Grading of Recommendations Assessment, Development and Evaluation
INR	International normalized ratio
LMWH	Low-molecular-weight heparin
MHV	Mechanical heart valve
PCI	Percutaneous coronary intervention
PT	Prothrombin time
PTChP	Polskie Towarzystwo Chorób Płuc (Polish Respiratory Society)
TBLB	Transbronchial lung biopsy using forceps
TBLC	Transbronchial lung biopsy using cryoprobe
UHF	Unfractionated heparin
VKA	Vitamin K antagonists
VTE	Venous thromboembolism
