# Supplementary material for: Guidelines for Minimizing Bleeding Risk During Bronchoscopic Procedures
Source: Adv Respir Med. 2026 Jun 30;94(4):44. doi: 10.3390/arm94040044 (PMC13397931; doi:10.3390/arm94040044)
Supplement: Supplementary file 1 [file arm-94-00044-s001.zip › arm-4348594-supplementary.pdf]

Guidelines for minimizing bleeding risk during bronchoscopic procedures

Appendix No. 1 – detailed clinical questions

Question 1. In patients with abnormal platelet count, is it safe to perform bronchoscopy, BAL, EBB, EBUS-TBNA, TBLB, and TBLC? What is the minimum platelet count that allows these procedures to be performed safely?

1.1. Is it safe to perform bronchoscopy in patients with abnormal platelet count?
1.2. Is it safe to perform EBB in patients with abnormal platelet count?
1.3. Is it safe to perform EBUS-TBNA in patients with abnormal platelet count?
1.4. Is it safe to perform TBLB in patients with abnormal platelet count?
1.5. Is it safe to perform TBLC in patients with abnormal platelet count?
1.6. What is the minimum platelet count required to refer a patient for bronchoscopy?
1.7. What is the minimum platelet count required to refer a patient for EBB?
1.8. What is the minimum platelet count required to refer a patient for EBUS-TBNA?
1.9. What is the minimum platelet count required to refer a patient for TBLB?
1.10. What is the minimum platelet count required to refer a patient for TBLC?

**Safe – the risk of complications is not significantly different from that of patients without the given risk factor*

Question 2. In patients with abnormal INR and APTT, is it safe to perform bronchoscopy, BAL, EBB, EBUS-TBNA, TBLB, and TBLC? What are the maximum INR and APTT values that allow these procedures to be performed safely?

2.1. Is it safe to perform bronchoscopy in patients with abnormal INR and APTT values?
2.2. Is it safe to perform EBB in patients with abnormal INR and APTT values?
2.3. Is it safe to perform EBUS-TBNA in patients with abnormal INR and APTT values?
2.4. Is it safe to perform TBLB in patients with abnormal INR and APTT values?
2.5. Is it safe to perform TBLC in patients with abnormal INR and APTT values?
2.6. What are the upper APTT and INR limits considered safe for referring a patient for bronchoscopy?
2.7. What are the upper APTT and INR limits considered safe for referring a patient for EBB?
2.8. What are the upper APTT and INR limits considered safe for referring a patient for EBUS-TBNA?
2.9. What are the upper APTT and INR limits considered safe for referring a patient for TBLB?
2.10. What are the upper APTT and INR limits considered safe for referring a patient for TBLC?

The above questions refer to patients not taking: 1) warfarin or acenocoumarol

Question 3. In patients taking 1 or 2 antiplatelet agents, is it safe to perform bronchoscopy, BAL, EBB, EBUS-TBNA, TBLB, and TBLC?

3.1. Is it safe to perform bronchoscopy in patients taking aspirin for cardioprotection?
3.2. Is it safe to perform EBB in patients taking aspirin for cardioprotection?
3.3. Is it safe to perform EBUS-TBNA in patients taking aspirin for cardioprotection?
3.4. Is it safe to perform TBLB in patients taking aspirin for cardioprotection?
3.5. Is it safe to perform TBLC in patients taking aspirin for cardioprotection?
3.6. Is it safe to perform bronchoscopy in patients taking aspirin for cardioprotection and a second antiplatelet drug (clopidogrel, prasugrel, or ticagrelor)?
3.7. Is it safe to perform EBB in patients taking aspirin for cardioprotection and a second antiplatelet drug (clopidogrel, prasugrel, or ticagrelor)?
3.8. Is it safe to perform EBUS-TBNA in patients taking aspirin for cardioprotection and a second antiplatelet drug (clopidogrel, prasugrel, or ticagrelor)?
3.9. Is it safe to perform TBLB in patients taking aspirin for cardioprotection and a second antiplatelet drug (clopidogrel, prasugrel, or ticagrelor)?
3.10. Is it safe to perform TBLC in patients taking aspirin for cardioprotection and a second antiplatelet drug (clopidogrel, prasugrel, or ticagrelor)?

**Cardioprotective dose of aspirin defined as ≤150 mg*

Question 4. In patients taking oral anticoagulants, is it safe to perform bronchoscopy, BAL, EBB, EBUS-TBNA, TBLB, or TBLC? If not, when should anticoagulation be discontinued before the procedure, and when should it be resumed afterward?

4.1. Is it safe to perform bronchoscopy in patients taking a VKA?
4.2. Is it safe to perform EBB in patients taking a VKA?
4.3. Is it safe to perform EBUS-TBNA in patients taking a VKA?
4.4. Is it safe to perform TBLB in patients taking a VKA?
4.5. Is it safe to perform TBLC in patients taking a VKA?
4.6. What is the recommended strategy for safely discontinuing a VKA before bronchoscopy?
4.7. What is the recommended strategy for safely discontinuing a VKA before EBB?
4.9. What is the recommended strategy for safely discontinuing a VKA before EBUS-TBNA?
4.10. What is the recommended strategy for safely discontinuing a VKA before TBLB?
4.11. What is the recommended strategy for safely discontinuing a VKA before TBLC?
4.12. Is it safe to perform bronchoscopy in patients taking a DOAC?
4.13. Is it safe to perform EBB in patients taking a DOAC?
4.14. Is it safe to perform EBUS-TBNA in patients taking a DOAC?

4.15. Is it safe to perform TBLB in patients taking a DOAC?
4.16. Is it safe to perform TBLC in patients taking a DOAC?
4.17. How many days before bronchoscopy should apixaban be discontinued to ensure procedural safety?
4.18. How many days before EBB should apixaban be discontinued to ensure procedural safety?
4.19. How many days before EBUS-TBNA should apixaban be discontinued to ensure procedural safety?
4.20. How many days before TBLB should apixaban be discontinued to ensure procedural safety?
4.21. How many days before TBLC should apixaban be discontinued to ensure procedural safety?
4.22. How many days before bronchoscopy should rivaroxaban be discontinued to ensure procedural safety?
4.23. How many days before EBB should rivaroxaban be discontinued to ensure procedural safety?
4.24. How many days before EBUS-TBNA should rivaroxaban be discontinued to ensure procedural safety?
4.25. How many days before TBLB should rivaroxaban be discontinued to ensure procedural safety?
4.26. How many days before TBLC should rivaroxaban be discontinued to ensure procedural safety?
4.27. How many days before bronchoscopy should dabigatran be discontinued to ensure procedural safety?
4.28. How many days before EBB should dabigatran be discontinued to ensure procedural safety?
4.29. How many days before EBUS-TBNA should dabigatran be discontinued to ensure procedural safety?
4.30. How many days before TBLB should dabigatran be discontinued to ensure procedural safety?
4.31. How many days before TBLC should dabigatran be discontinued to ensure procedural safety?
4.32. In patients receiving DOACs, does a management strategy involving temporary discontinuation of the DOAC and bridging with prophylactic-dose LMWH allow for the safe performance of bronchoscopy, EBB, TBLB, and EBUS-TBNA?

Question 5. Is it safe to perform bronchoscopy, BAL, EBB, EBUS-TBNA, TBLB, and TBLC in patients receiving prophylactic or therapeutic low-molecular-weight heparin (LMWH), and if not, when should LMWH be withheld before the procedure and when should it be restarted afterward?
5.1. Is it safe to perform bronchoscopy in patients receiving prophylactic-dose LMWH?

5.2. Is it safe to perform EBB in patients receiving prophylactic-dose LMWH?
5.3. Is it safe to perform EBUS-TBNA in patients receiving prophylactic-dose LMWH?
5.4. Is it safe to perform TBLB in patients receiving prophylactic-dose LMWH?
5.5. Is it safe to perform TBLC in patients receiving prophylactic-dose LMWH?
5.6. Is it safe to perform bronchoscopy in patients receiving therapeutic-dose LMWH?
5.7. Is it safe to perform EBB in patients receiving therapeutic-dose LMWH?
5.8. Is it safe to perform EBUS-TBNA in patients receiving therapeutic-dose LMWH?
5.9. Is it safe to perform TBLB in patients receiving therapeutic-dose LMWH?
5.10. Is it safe to perform TBLC in patients receiving therapeutic-dose LMWH?
5.11. On the day of bronchoscopy, when should LMWH be administered if used at prophylactic or therapeutic doses?
5.12. How long before bronchoscopy with biopsy should LMWH be discontinued when administered at prophylactic or therapeutic doses?
5.13. When can LMWH in a prophylactic or therapeutic dose be resumed after bronchoscopy with biopsy?

Appendix 2. Literature search

A literature search was conducted in PubMed and EMBASE in March 2025. The search strategy combined free-text terms with controlled vocabulary from the MeSH and EMTREE thesauri, without any language restrictions. We included various types of primary publications and also publications such as guidelines and secondary studies (systematic reviews, meta-analyses, or review articles). The database search was supplemented by manual screening of reference lists from included publications (including identified guidelines) and relevant scientific society websites. A total of 3297 records were retrieved from structured search in PubMed and EMBA, and handsearch (Appendix Figure 1). During the process of guidelines development, two panel members (cardiologist and hematologist) have proposed some additional publications which were included in the final list od references. Despite no language filter was used during the search process, finally two identified documents available only in Chinese were excluded.

A. PubMed strategy:

i) (thrombocytopenia*[Title/Abstract] OR "anticoagulant agent" [Title/Abstract] OR anticoagulation[MeSH Terms] OR thrombocytopeni*[Title/Abstract] OR thrombocytopaeni*[Title/Abstract] OR apt[Title/Abstract] OR "blood clotting disorder"[Title/Abstract] OR "haematological abnormalities" [Title/Abstract] OR "prothrombin time" [Title/Abstract] OR pt[Title/Abstract] OR "kaolin clotting time" [Title/Abstract] OR "activated partial thromboplastin time" [Title/Abstract] OR "anticoagulant agent" [Title/Abstract] OR anticoagu*[Title/Abstract] OR "coagulation abnormalities" [Title/Abstract]) AND (bronchoscopy[Title/Abstract] OR "bronchial mucosa biopsy"[Title/Abstract] OR "transbronchial lung biopsy"[Title/Abstract] OR "transbronchial biopsy"[Title/Abstract] OR ("ultrasound-guided lymph nod*"[Title/Abstract] AND biops*[Title/Abstract]) OR "endobronchial ultrasound guided transbronchial needle aspiration"[Title/Abstract] OR ebus[Title/Abstract] OR tblb[Title/Abstract] OR "endobronchial ultrasonography"[Title/Abstract] OR "endoscopic ultrasound guided fine needle biopsy"[Title/Abstract] OR "Bronchoscopy"[Mesh] OR "Biopsy"[Mesh] OR "Endoscopic Ultrasound-Guided Fine Needle Aspiration"[Mesh] OR "Biopsy, Fine-Needle"[Mesh])

ii) ("Anticoagulants"[Mesh] OR "Antithrombins"[Mesh] OR "Heparin, Low-Molecular-Weight" [Mesh] OR "Heparin"[Mesh] OR "Dabigatran"[Mesh] OR "Rivaroxaban"[Mesh] OR "Factor Xa Inhibitors"[Pharmacological Action] OR "new oral anticoagulant*" [Title/Abstract] OR "direct oral anticoagulant" [Title/Abstract]

OR "non vitamin k antagonist oral anticoagulant" [Title/Abstract] OR "factor iia" [Title/Abstract] OR “thrombin inhibitor" [Title/Abstract] OR dabigatran[Title/Abstract] OR rivaroxaban[Title/Abstract] OR heparin[Title/Abstract] OR "low molecular weight heparin" [Title/Abstract] OR enoxaparin[Title/Abstract] OR dalteparin[Title/Abstract] OR tedelparin[Title/Abstract] OR "anticoagulant agent" [Title/Abstract]) AND (bronchoscopy[Title/Abstract] OR "bronchial mucosa biopsy"[Title/Abstract] OR "transbronchial lung biopsy"[Title/Abstract] OR "transbronchial biopsy"[Title/Abstract] OR ("ultrasound-guided lymph nod*"[Title/Abstract] AND biops*[Title/Abstract]) OR "endobronchial ultrasound guided transbronchial needle aspiration"[Title/Abstract] OR ebus[Title/Abstract] OR tblb[Title/Abstract] OR "endobronchial ultrasonography"[Title/Abstract] OR "endoscopic ultrasound guided fine needle biopsy"[Title/Abstract] OR "Bronchoscopy"[Mesh] OR "Biopsy"[Mesh] OR "Endoscopic Ultrasound-Guided Fine Needle Aspiration"[Mesh] OR "Biopsy, Fine-Needle"[Mesh])

iii) (Anticoagulants[Mesh] OR coumarins[Mesh] OR "vitamin k antagon*"[Title/Abstract] OR VKA[Title/Abstract] OR anticoagula*[Title/Abstract] OR "anti-coagula*"[Title/Abstract] OR warfarin[Title/Abstract] OR dindevan[Title/Abstract] OR Jantoven[Title/Abstract] OR Marevan[Title/Abstract] OR Lawarin[Title/Abstract] OR Waran[Title/Abstract] OR Warfant[Title/Abstract] OR sinthrome[Title/Abstract] OR sintrom[Title/Abstract] OR marcumar[Title/Abstract] OR falithrom[Title/Abstract] OR acenocoumarol[Title/Abstract] OR aldocumar[Title/Abstract] OR brodifacoum[Title/Abstract] OR clorindione[Title/Abstract] OR coumatetralyl[Title/Abstract] OR dicoumarol[Title/Abstract] OR diphenadione[Title/Abstract] OR flocoumafen[Title/Abstract] OR phenindione[Title/Abstract] OR phenprocoumon[Title/Abstract] OR pindone[Title/Abstract] OR superwarfarin[Title/Abstract] OR tecarfarin[Title/Abstract] OR tioclomarol[Title/Abstract] OR anisindione[Title/Abstract] OR chlorophacinone[Title/Abstract] OR diphacinone[Title/Abstract] OR fluindione[Title/Abstract]) AND (bronchoscopy[Title/Abstract] OR (bronchial[Title/Abstract] AND mucosal[Title/Abstract] AND biopsy[Title/Abstract]) OR (transbronchial[Title/Abstract] AND lung[Title/Abstract] AND biopsy[Title/Abstract]) OR (Transbronchial[Title/Abstract] AND biopsy[Title/Abstract]) OR TBLB[Title/Abstract] OR (Ultrasound-guided[Title/Abstract] AND "lymph node" [Title/Abstract] AND biopsy[Title/Abstract]) OR EBUS-FNA[Title/Abstract] OR "endobronchial ultrasound-guided transbronchial needle aspiration"[Title/Abstract] OR " EBUS-TBNA"[Title/Abstract] OR ("endobronchial ultrasonography"[Title/Abstract] AND "endoscopy ultrasound-guided"[Title/Abstract] AND "fine-needle

aspiration"[Title/Abstract]) OR EUS-FNA[Title/Abstract] OR ("endoscopic ultrasound"[Title/Abstract] AND "EBUS scope-guided fine-needle aspiration"[Title/Abstract]) OR EUS-B-FNA[Title/Abstract] OR ("endoscopic ultrasound"[Title/Abstract] AND "EBUS bronchoscope"[Title/Abstract]) OR ("transesophageal endoscopic"[Title/Abstract] AND "ultrasound-guided needle aspiration"[Title/Abstract]) OR ("transesophageal endoscopic"[Title/Abstract] AND "ultrasound-guided fine-needle aspiration"[Title/Abstract]) OR "Transbronchial Lung Cryobiopsy" [Title/Abstract]) OR ("Endobronchial ultrasound guided"[Title/Abstract] AND "transbronchial lymph node cryobiopsy"[Title/Abstract]))

B. EMBASE strategy

i) ('thrombocytopenia'/exp OR 'anticoagulant agent'/exp OR 'anticoagulation'/exp OR thrombocytopeni*:ti,ab,kw OR thrombocytopaeni*:ti,ab,kw OR 'apt':ti,ab,kw OR 'blood clotting disorder':ti,ab,kw OR 'haematological abnormalities':ti,ab,kw OR 'prothrombin time':ti,ab,kw OR 'pt':ti,ab,kw OR 'kaolin clotting time':ti,ab,kw OR 'activated partial thromboplastin time':ti,ab,kw OR 'anticoagulant agent':ti,ab,kw OR anticoagu*:ti,ab,kw OR 'coagulation abnormalities':ti,ab,kw) AND ('bronchoscopy'/exp OR 'transbronchial biopsy'/exp OR 'ultrasound guided fine needle aspiration'/exp OR 'endobronchial ultrasonography'/exp OR 'endoscopic ultrasound guided fine needle biopsy'/exp OR 'bronchoscopy':ti,ab,kw OR 'bronchial mucosa biopsy':ti,ab,kw OR 'transbronchial lung biopsy':ti,ab,kw OR 'transbronchial biopsy':ti,ab,kw OR ('ultrasound-guided lymph nod*':ti,ab,kw AND biops*:ti,ab,kw) OR 'endobronchial ultrasound guided transbronchial needle aspiration':ti,ab,kw OR 'endoscopic ultrasound using the ebus scope-guided fine-needle aspiration':ti,ab,kw OR tblb:ti,ab,kw OR 'endobronchial ultrasonography':ti,ab,kw OR 'endoscopic ultrasound guided fine needle biopsy':ti,ab,kw)

ii) ('antithrombotic agent'/exp OR 'new oral anticoagulant'/exp OR 'novel oral anticoagulant'/exp OR 'direct oral anticoagulant'/exp OR 'non vitamin k antagonist oral anticoagulant'/exp OR 'blood clotting factor 10a inhibitor'/exp OR 'thrombin inhibitor'/exp OR 'low molecular weight heparin'/exp OR 'heparin'/exp OR 'dabigatran'/exp OR 'anticoagulant agent'/exp OR 'rivaroxaban'/exp OR 'apixaban'/exp OR 'edoxaban'/exp OR 'enoxaparin'/exp OR 'dalteparin'/exp OR 'tedelparin'/exp OR 'antithrombotic agent':ti,ab,kw OR 'new oral anticoagulant*':ti,ab,kw OR 'direct oral anticoagulant':ti,ab,kw OR 'non vitamin k antagonist oral anticoagulant':ti,ab,kw OR 'blood clotting factor 10a inhibitor':ti,ab,kw OR 'factor iia':ti,ab,kw OR 'factor specific oral anticoagulants':ti,ab,kw OR 'thrombin inhibitor':ti,ab,kw OR 'dabigatran':ti,ab,kw OR 'rivaroxaban':ti,ab,kw OR 'apixaban':ti,ab,kw OR 'edoxaban':ti,ab,kw OR 'heparin':ti,ab,kw OR 'low molecular weight heparin':ti,ab,kw OR

'enoxaparin':ti,ab,kw OR 'dalteparin':ti,ab,kw OR 'tedelparin':ti,ab,kw OR 'anticoagulant agent':ti,ab,kw) AND ('bronchoscopy'/exp OR 'transbronchial biopsy'/exp OR 'ultrasound guided fine needle aspiration'/exp OR 'endobronchial ultrasonography'/exp OR 'endoscopic ultrasound guided fine needle biopsy'/exp OR 'bronchoscopy':ti,ab,kw OR 'bronchial mucosa biopsy':ti,ab,kw OR 'transbronchial lung biopsy':ti,ab,kw OR 'transbronchial biopsy':ti,ab,kw OR ('ultrasound-guided lymph nod*':ti,ab,kw AND biops*:ti,ab,kw) OR 'endobronchial ultrasound guided transbronchial needle aspiration':ti,ab,kw OR 'endoscopic ultrasound using the ebus scope-guided fine-needle aspiration':ti,ab,kw OR tblb:ti,ab,kw OR 'endobronchial ultrasonography':ti,ab,kw OR 'endoscopic ultrasound guided fine needle biopsy':ti,ab,kw)

iii) ('anticoagulant agent'/exp OR 'coumarin derivative'/exp OR k NEAR/3 antagon* OR vka:ti,ab,kw OR 'vitamin k antagonist':ti,ab,kw OR 'anticoagulant agent':ti,ab,kw OR 'anti-coagulant agent':ti,ab,kw OR 'warfarin'/exp OR warfarin:ti,ab,kw OR 'phenindione':ti,ab,kw OR 'acenocoumarol':ti,ab,kw OR 'phenprocoumon':ti,ab,kw OR 'warfarin':ti,ab,kw OR 'brodifacoum':ti,ab,kw OR 'clorindione':ti,ab,kw OR 'coumatetralyl'/exp OR 'coumatetralyl':ti,ab,kw OR 'dicoumarol':ti,ab,kw OR 'diphenadione':ti,ab,kw OR 'diphenadione'/exp OR 'flocoumafen':ti,ab,kw OR 'flocoumafen'/exp OR 'phenindione':ti,ab,kw OR 'phenindione'/exp OR 'phenprocoumon':ti,ab,kw OR 'pindone':ti,ab,kw OR 'coumadin':ti,ab,kw OR 'jantoven':ti,ab,kw OR 'sintrom':ti,ab,kw OR 'syncumar':ti,ab,kw OR 'superwarfarin':ti,ab,kw OR 'tecarfarin':ti,ab,kw OR tioclomarol:ti,ab,kw OR 'anisindione'/exp OR 'anisindione':ti,ab,kw OR 'chlorophacinone':ti,ab,kw OR 'chlorophacinone'/exp OR 'diphenadione':ti,ab,kw OR 'diphenadione'/exp OR 'fluindione':ti,ab,kw OR 'fluindione'/exp) AND (bronchoscopy OR (bronchial AND mucosal AND biops*) OR (Transbronchial AND lung AND biopsy) OR (Transbronchial AND biopsy) OR TBLB OR ((Ultrasound-guided AND lymph nod*) AND biopsy) OR EBUS-FNA OR (endobronchial ultrasound-guided transbronchial needle aspiration) OR “EBUS-TBNA” OR (“endobronchial ultrasonography” AND “endoscopy ultrasound-guided fine-needle aspiration”) OR “EUS-FNA” OR (endoscopic ultrasound using the EBUS scope-guided fine-needle aspiration) OR “EUS-B-FNA” OR (endoscopic ultrasound using the EBUS bronchoscope) OR (transesophageal endoscopic ultrasound-guided needle aspiration) OR (transesophageal endoscopic ultrasound-guided fine-needle aspiration) OR (Transbronchial Lung Cryobiopsy) OR (Endobronchial ultrasound guided transbronchial lymph node cryobiopsy))

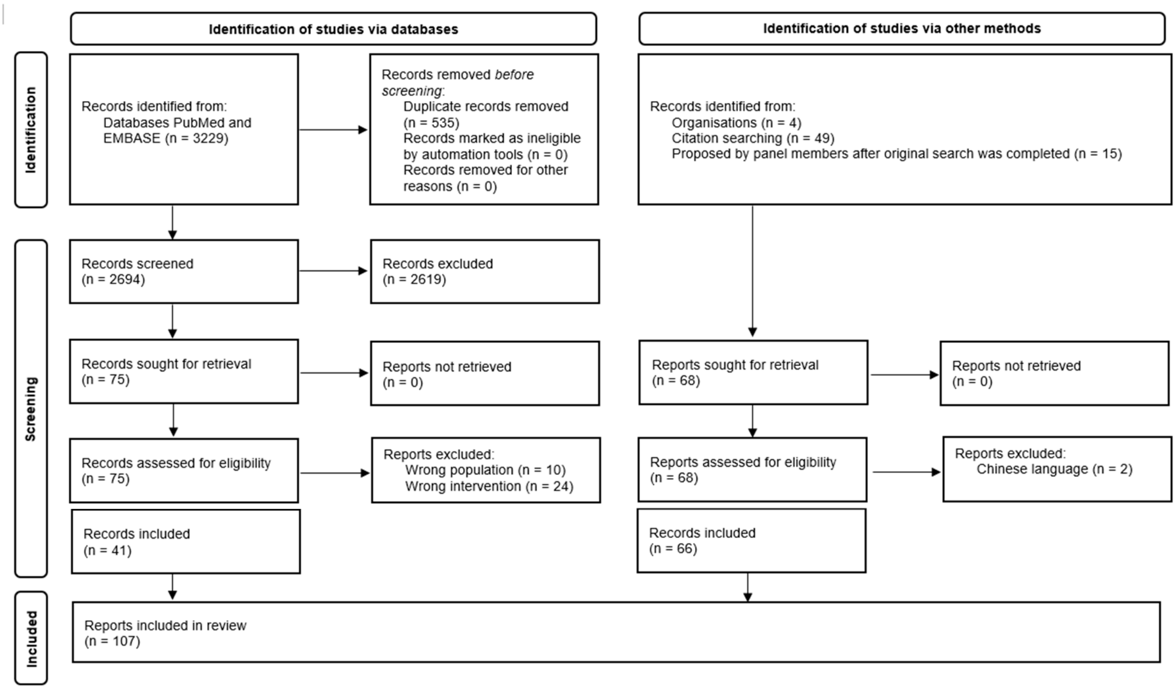

Figure S1. Flowchart illustrating the selection process of reports.
